# Should we stop using the determination of central venous pressure as a way to estimate cardiac preload?

**Published:** 2012-06-30

**Authors:** Johann Smith Cerón Arias, Manuel Felipe Muñoz Nañez

**Affiliations:** a Universidad del Cauca Email johanceron@gmail.com; b Anesthesiologist Hospital Universitario del Valle Colombia Email felpemunoz99@hotmail.com; c Anesthesiologist IntensiveCareUnit Clínica La Estancia Popayán Colombia

**Keywords:** Central venous pressure, blood volume, stroke volume, pulse pressure change, global end-diastolic volume, diastolic area

## Abstract

**Introduction:**

The determination of the values of central venous pressure has long been used as a guideline for volumetric therapy in the resuscitation of the critical patient, but the performance of such parameter is currently being questioned as an effective measurement of cardiac preload. This has aroused great interest in the search for more accurate parameters to determine cardiac preload and a patient's blood volume.

**Goals and Methods:**

Based on literature currently available, we aim to discuss the performance of central venous pressure as an effective parameter to determine cardiac preload.

**Results and Conclusion:**

Estimating variables such as end-diastolic ventricular area and global end-diastolic volume have a better performance than central venous pressure in determining cardiac preload. Despite the best performance of these devices, central venous pressure is still considered in our setting as the most practical and most commonly available way to assess the patient's preload. Only dynamic variables such as pulse pressure change are superior in determining an individual's blood volume.

## Abstract (es)

**Introducción:**

La determinación de los valores de presión venosa central han servido por mucho tiempo como guía a la terapia volumétrica en la reanimación del paciente critico, sin embargo su rendimiento como medida eficaz de la precarga cardiaca actualmente es muy discutido, suscitando un gran interés por encontrar parámetros que se acerquen mas a la determinación de la precarga cardiaca o del estado de volemia del paciente.

**Objetivos y Metodos:**

Con base a la literatura actual disponible, pretendemos discutir el rendimiento de la presión venosa central como parámetro eficaz en la determinación de la precarga cardiaca.

**Resultados y Conclusión:**

La estimación de variables como el área ventricular de fin de diástole y el volumen global de fin de diástole poseen un mejor rendimiento que la presión venosa central en la determinación de la precarga cardiaca. A pesar del mejor desempeño de estos dispositivos la presión venosa central sigue siendo en nuestro medio la forma más práctica y disponible a la cabecera del paciente para valorarla.Solo, variables dinámicas como la variación de la presión de pulso son superiores en la capacidad de predecir la respuesta al volumen.

## Introduction

Preload plays an important role in determining cardiac output and, hence, its optimization improves reanimation of patients in critical state^1^.

An increasing number of published studies demonstrate how the determination of cardiac filling pressure through a central venouscatheter is affected by multiple intra- and extra-cardiac factors that can lead measurement and interpretation error^2^; thereby, this has led to searching for other parameters closer to the determination of preload, like the end-diastolic area currently considered the Gold Standard^3^ and the filling volumes through systems like trans-cardiopulmonary thermodilution^4^. However, given that these are static measurements, these fall short in predicting the response to the liquid infusion, yielding to new clinical variables like the pulse pressure variability, which integrates preload and its response to it.

## Defining preload

Preload has long been considered an estimation of volemia, but although volemia is one of the determinants of preload, these are two different terms. Thereby, to start the development of our discussion, we must clarify these concepts.

Preload is defined as the change of longitude of the myocardial fiber generated by the force exerted by the entry of a given amount of blood during the diastole moment^5^. Volemia refers to the circulating blood volume of an individual's total economy at a given moment^6^. Now, with these concepts cleared and centered on our discussion, we must ask ourselves: can pressure measurements in a central vein effectively estimate preload or even an individual's volemia?

## Central venous pressure as an estimation of preload

It is acceptable to think that if we determine the cardiac filling pressure, we are interpreting the force with which blood reaches the heart and, hence, the preload, but the volume the heart can contain at the moment of diastole is initially approached with a parameter that convincingly determines the pressure readings generated by such on its walls, and it is the distensibility^7^ of the myocardial fiber, which establishes the longitude such can reach (preload) or the volume that it can accommodate; thus, we cannot assume that pressures generated in the heart are always directly proportional to the volume contained, especially when not considering the degree of distensibility of the myocardial fiber because of individual variability or due to different pathological states affecting the cardiac muscle. A study by Kumar *et al*.,^8^ reveals this phenomenon when finding a scarce relationship of the values of central venous pressure and its variation with the systolic volume in healthy individuals.

Other factors added to the inadequate interpretation and/or reading are the position of the patient and the juxta-cardiac pressure transmitted to the central catheter and which are influenced by: pleural pressure, tele-expiratory positive pressure, intra-pericardial pressure, and intra-abdominal pressure among others^9^, which must be kept in mind when interpreting the values yielded and which must also be accompanied by a careful physiopathological analysis of the patient's status. Therefore, although the values of cardiac filling pressure continue being useful in the clinical interpretation of preload in our setting, essentially because of their availability, when such do not have an adequate correlation between its values and the volumetric response there is the need to search for other devices to better estimate preload by measuring variables like the end-diastolic ventricular area via ultrasound or the determination of the cardiac and intrathoracic filling volumes through trans-cardiopulmonary thermodilution^2^, which although better related to the preload concept and to the response to it (systolic volume), have limitations like not being able to have it at the patient's bedside 24 hours per day for the first and not having said technology in our country for the latter. These factors have converged on the central venous pressure still being used in our setting as an estimation of the force with which the blood reaches the heart, analyzing the tendency over time and taking the necessary care to diminish error in its interpretation, subjecting its reading to strict clinical and physiopathological judgment.

## Central venous pressure as an estimation of volemia or response To the administration of volume

Currently, clinical interest is aimed at predicting a patient's response upon the load of a given volume (response to preload), more than to its static determination^1^ and, thus, be able to predict which patients will improve their cardiac output with the liquid infusion or which patients will be exposed to volumetric overload because of these.

**Figure 1:**
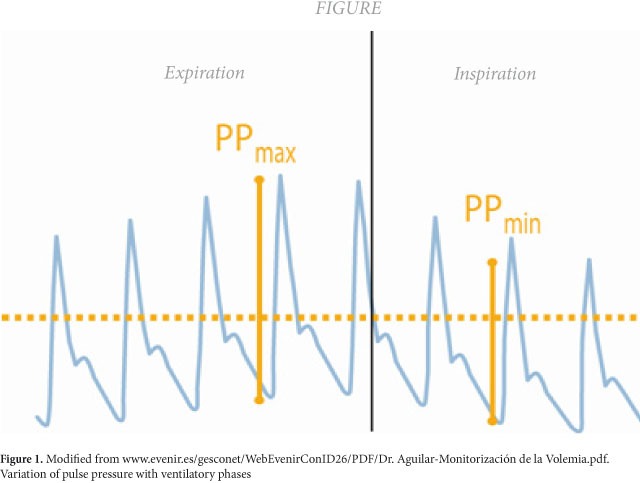
Modified from www.evenir.es/gesconet/WebEvenirConID26/PDF/Dr. Aguilar-Monitorización de la Volemia.pdf. Variation of pulse pressure with ventilatory phases

For patients under mechanical ventilation, the magnitude of changes in the systolic volume of the left ventricle given by the ventilatory phase they are in is proposed as parameters to detect the response to the administration of volume^10^. Availing of the heart-lung interaction, if we observe closely the pulse pressure dicrotic wave generated by a peripheral artery in patients on mechanical ventilation, we will note a variation in its contour according to the phase of the current ventilatory cycle^2^ ^,^ ^3^ ^,^ ^10^; this is how during inspiration, the wave tends to rise and during the expiratory phase it is depressed (Fig. 1). This difference in its peaks has been proposed as a predictor of the response to the variation of the systolic volume upon increased preload^2^ ^,^ ^3^ ^,^ ^10^. 

**Figure 2:**
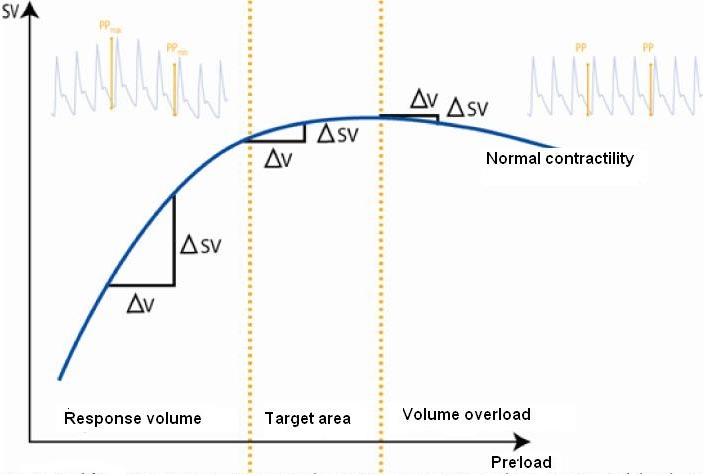
Modified from www.evenir.es/gesconet/WebEvenirConID26/PDF/Dr. Aguilar-Monitorización de la Volemia.pdf. Ventricular function curve by Frank-Starling and its relation to the pulse pressure variation. AV: variation volume. ASV: variation of systolic volume

The relation between the variations and their response to preload respond to the relation Frank and Starling reflected in their ventricular function curve and which is comprised of two phases^5^ (Fig. 2). When the patient "moves" in phase 1 of the curve, preload is directly proportional to the systolic volume; the variations of pulse are broad, indicating that the patient is dependent on the preload or that the patient will respond to the volumen^2^ ^,^ ^3^ ^,^ ^10^ (responder or dependent on volume), while if the patient is moved to phase 2 of the Starling curve the variations in pulse pressure are minimal, indicating that the patient will not respond to the liquid infusion or that what is probably required is to strengthen cardiac inotropism to increase cardiac output and avoid an overload of liquids that can lead to pulmonary edema^2^ ^,^ ^3^ ^,^ ^10^ (non-responder or not dependent on volume).

This is why determining the state of volemia that was being carried out based on the tendency over time of static variables like central venous pressure does not work, given that it does not integrate the relation between preload and its response (effective systolic volume). Marik* et al*.,^11^ in a Meta-analysis including 188 ICU patients and where 2,500 measurements of central venous pressure were conducted concluded that measurements of central venous pressure and their changes are not useful as estimations of volemia.

Hence, when determining dynamic parameters of response to the volume infusion, it is more interesting for the clinician because it becomes a practical tool that integrates the two factors conditioning the systolic volume or a patient's effective state of volemia, such as preload and the integrity of the myocardial fiber. Validation of these parameters has taken place in a variety of patients under different pathological conditions like acute respiratory distress syndrome (ARDS), septic shock, neurosurgical patients, and pulmonary transplant with a positive predictive value of 94% and a negative predictive value of 96%^4^ ^,^ ^12^ ^-^ ^15^.

Nevertheless, it is absolutely necessary to comply with the following recommendations for an optimal measurement:

1. Patients must be connected to 100% controlled Mechanical Ventilation.

2. The flow volume must be at least 7 mL/kg and less than 10 mL/kg.

3. Tele-expiratory pressure less than 10 cm of water.

4. Remain in a regular sinus rhythm for measurement. 

On the contrary, the measurement parameter is not reliable.

## Conclusion

The estimation of variables like ventricular area and global end-diastolic volume offer better performance than the central venous pressure in determining cardiac preload. But, is spite of the better performance of these variables, the central venous pressure continues being the most widely manner to assess preload in our setting due to its convenience and availability at the patient's bedside.

However, current clinical falls on predicting response to fluid therapy and only dynamic variables like variation of pulse pressure are superior and, thus more effective in predicting the response of critical patients under mechanical ventilation to the volume challenge. Thereby, when using the central venous pressure as an estimation of preload we must eliminate factors of error that can lead us to a false reading of such like an inadequate position of the patient and keep in mind other factors like the analysis of artifacts that cause interpretation errors like juxta-cardiac pressures that can be transmitted to the measuring device; additionally, noting its tendency over time and evaluating other clinical signs related to adequate organ perfusion.
